# Predictors of Insulin Resistance in Children versus Adolescents with Obesity

**DOI:** 10.1155/2017/3793868

**Published:** 2017-12-10

**Authors:** Yvette E. Lentferink, Marieke A. J. Elst, Catherijne A. J. Knibbe, Marja M. J. van der Vorst

**Affiliations:** ^1^Department of Pediatrics, St. Antonius Hospital, Nieuwegein/Utrecht, Postbus 2500, 3430 EM Nieuwegein, Netherlands; ^2^Department of Clinical Pharmacy, St. Antonius Hospital, Nieuwegein/Utrecht, Postbus 2500, 3430 EM Nieuwegein, Netherlands

## Abstract

**Introduction:**

Obesity is a risk factor to develop metabolic syndrome (MetS) and type 2 diabetes mellitus (T2DM). Insulin resistance (IR) plays a major part in both. With increasing incidence of childhood obesity, this retrospective study aimed to identify predictors of IR in children/adolescents with obesity to optimize screening for IR.

**Method:**

Patients aged ≥ 2–≤ 18 years with obesity (BMI-SDS > 2.3) were included. IR was defined as HOMA-IR ≥ 3.4, and MetS if ≥3 of the following criteria were present: waist circumference and blood pressure ≥ 95th age percentile, triglycerides ≥ 1.7 mmol/l, HDL < 1.03 mmol/l, and fasting plasma glucose ≥ 5.6 mmol/l.

**Results:**

In total, 777 patients were included. Of the 306 children, 51, 38, and 0 were diagnosed with IR, MetS, and T2DM, respectively. Of the 471 adolescents, 223, 95, and 0 were diagnosed with IR, MetS, and T2DM, respectively. In the multivariable regression model, BMI-SDS, preterm birth, and Tanner stage were associated with IR in children (6.3 (95% CI 1.3–31.1), 5.4 (95% CI 1.4–20.5), 2.2 (95% CI 1.0–4.8)), and BMI-SDS and waist circumference in adolescents (4.0 (95% CI 1.7–9.2), 3.7 (95% CI 1.5–9.4)).

**Conclusion:**

Different IR predictors were observed in children/adolescents with obesity. These predictors can be used to optimize screening for IR in pediatric populations.

## 1. Introduction

Obesity is a major health problem with an expanding burden on society, not only due to a rising prevalence in both the developed and developing world but also because of the onset at younger age [1–3]. As a consequence, associated complications such as metabolic syndrome (MetS), type 2 diabetes mellitus (T2DM), cardiovascular diseases, respiratory illnesses, and psychosocial problems are now more frequently seen in pediatric populations [4, 5].

Insulin resistance (IR) is associated with obesity due to lipolytic effects of adipocytes, leading to large amounts of free fatty acids and impaired secretion of adipokines, both involved in the modulation of insulin sensitivity [6, 7]. IR is usually the first sign of a disturbed glucose metabolism. In case of IR, insulin production by the pancreatic beta cells is increased, causing hyperinsulinemia [6, 8, 9]. Failure of this compensatory response leads to impaired glucose tolerance (IGT) and eventually T2DM [8, 10]. Apart from being a risk factor for T2DM, IR has also been recognized as an independent risk factor for development of cardiovascular diseases by potentiating the onset of dyslipidaemia [6, 11]. Moreover, IR is frequently associated with MetS, whose components are (central) obesity, high blood pressure, high triglycerides, low HDL, and impaired fasting plasma glucose (FPG) [8, 12].

The gold standard to determine IR is the euglycemic-hyperinsulinemic clamp study. This invasive method is not routinely used in daily clinical practice; instead, surrogate measures are used. The measures differ in methods and cutoff values and are used concurrently, causing high variation in reported prevalence rates [13, 14]. The Homeostasis Model for the Assessment of Insulin Resistance (HOMA-IR) is the most commonly used method in daily practice [14]. In the pediatric population, IR is commonly seen in adolescents, as transient insulin resistance occurs during puberty due to high circulating levels of growth hormone [8, 15, 16]; however, IR is also observed before puberty [8]. A higher prevalence of IR is seen in adolescents with obesity irrespective of puberty [17], which implicates a relation between IR and obesity, as described above [6–8]. Recently, IR is increasingly observed in children with obesity [18, 19]. While this can be a consequence of the rising prevalence of obesity at younger age, recent studies also hypothesized other risk factors, such as preterm birth, low birth weight, absence of breastfeeding, and family history of diabetes and obesity [4, 18–20].

Identifying predictors of IR in the obese pediatric population is important as they can be used as a screening tool for those at risk for complications such as MetS and T2DM. Furthermore, since different predictors of IR are supposed in children and adolescents with obesity, it is important to distinguish between these two age groups. Thus far, studies comparing predictors of IR in children and adolescents with obesity are limited. Therefore, the aim of the current retrospective study is to identify predictors of IR in a cohort of children and adolescents with obesity, to optimize screening for IR.

## 2. Method

### 2.1. Study Design and Subjects

This retrospective cross-sectional observational study was approved by the Medical Ethical Committee of the St. Antonius Hospital, Nieuwegein/Utrecht, the Netherlands (W15.038). Need for written informed consent was waived as only data obtained during routine clinical care were used and analyzed anonymously. This implies that no additional blood samples were taken for this study. Patients who visited the pediatric outpatient clinic of the St. Antonius Hospital, between 2006 and 2014, with the DBC code “adiposity” (“Diagnose Behandeling Combinatie,” abbreviated to “DBC” in Dutch) were considered. Inclusion criteria were age ≥ 2–≤ 18 years, obesity (defined as BMI-SDS > 2.3), and available laboratory results on fasting plasma glucose (FPG) and fasting plasma insulin (FPI). Patients with weight-affecting disorders were excluded.

### 2.2. Measurements

From (electronic) medical records, patient demographics and anthropometric measurements, that is, date of birth, gender, ethnicity, term of birth, birth weight, infant feeding, length, weight, waist circumference, blood pressure, and stage of puberty, were retrieved. Moreover, laboratory results of blood samples, taken for routine clinical care, for FPG, FPI, high-density lipoprotein (HDL), low-density protein (LDL), triglycerides, and total cholesterol (TC) and family history of obesity, T2DM, dyslipidaemia, hypertension, and cardiovascular diseases (CVDs) were collected.

Participants were classified as children if they were <10 years of age and as adolescents if they were ≥ 10 years of age. Ethnicity was classified as Caucasian or non-Caucasian. Term of birth was categorized into preterm (gestational age ≤ 37 weeks) and term or postterm (gestational age > 37 weeks). Birth weight was categorized into appropriate for gestational age (AGA) and small or large for gestational age (SGA ≤ 5th percentile/LGA ≥ 95th percentile), using reference values of the Dutch Perinatal Registry [21]. Infant feeding was divided into breastfeeding, defined as administered for at least 28 days, or formula feeding. All anthropometric measurements and laboratory results were obtained from the intake visit to the pediatric outpatient clinic. Weight and length were measured using a digital scale (Seca, Hamburg, Germany) with an accuracy of 0.05 kg and a digital stadiometer with a precision of 0.1 cm (DGI 250D, De Grood, Nijmegen, the Netherlands), respectively. Body mass index (BMI) and BMI standard deviation score (SDS) were calculated using the TNO growth calculator for professionals [22]. Waist circumference was measured midway, between the lowest rib and upper border of the iliac crest at the end of a gentle expiration, with a precision of 0.1 cm, using an inextensible tape with millimeter scale while the patient was in standing position. Waist circumference was categorized in normal or obese waist, based on Dutch reference values [23]. Stage of puberty was assessed by the attending physician, using Tanner stage, and categorized into prepubertal (T1), pubertal (T2–4), and postpubertal (T5). Blood pressure was measured in supine position from the right arm. Hypertension was defined as systolic and/or diastolic blood pressure ≥ 95th percentile for age [24].

IR was calculated using Homeostatic Model Assessment for Insulin Resistance (HOMA-IR) (FPG (mmol/L) × FPI (mU/L)/22.5) [25] and defined as HOMA-IR ≥ 3.4 [26]. MetS was defined as the presence of at least 3 of the following criteria: waist circumference ≥ 95th percentile for age [23], systolic blood pressure and/or diastolic blood pressure ≥ 95th percentile for age [24], triglycerides ≥ 1.7 mmol/l, HDL < 1.03 mmol/l, and fasting plasma glucose ≥ 5.6 mmol/l. Family history of obesity, T2DM, dyslipidaemia, hypertension, and CVDs were categorized as first degree, second degree, or no family affected.

### 2.3. Statistical Analysis

Statistical analysis was performed using IBM SPSS Statistics, version 24 (IBM SPSS Statistics, Chicago, IL, USA). Normally distributed continuous parameters were reported as mean ± standard deviation and nonparametric continuous parameters as median with range. Categorical data were expressed as frequencies with percentage. Student's *t*-test was used to compare normal distributed continuous variables and the Mann–Whitney *U* test for nonnormal distributed data. The chi-squared test was used to analyze differences in categorical variables and the one-way ANOVA or Kruskal–Wallis test in nonparametric data, when comparing more than two groups. Binary logistic regression was used to investigate the association between IR and BMI-SDS, Tanner stage, term of birth, birth weight, breastfeeding, and waist circumference, with adjustment for age, gender, and ethnicity. An *α*-level of 5% was considered significant for all statistical tests.

## 3. Results

**Figure 1:**
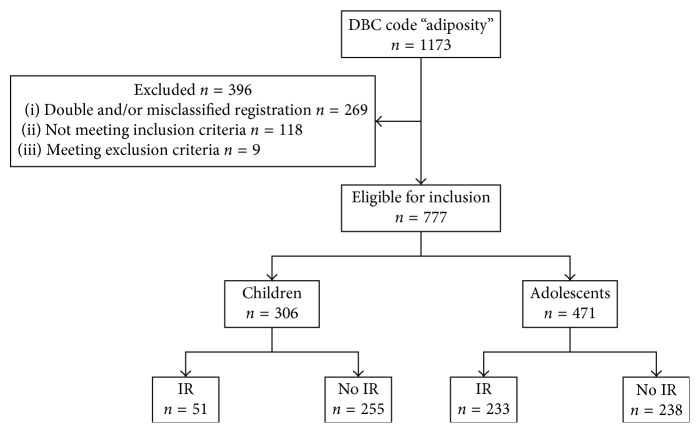
Flowchart of the study population.

Figure 1 shows that in total 1173 patients with obesity were considered for inclusion. For various reasons, 396 patients were excluded. In total, 777 patients were included, 306 children and 471 adolescents, of which 51 and 223 were diagnosed with IR, respectively.

Tables 1–3 show the demographics, anthropometric measurements, and laboratory results, respectively, of the included children and adolescents, with and without IR. Both children and adolescents with IR were significantly older and taller and had a higher weight and higher BMI-SDS (Tables 1 and 2). In addition, both children and adolescents had significantly higher triglycerides and lower HDL (Table 3). MetS was diagnosed significantly more in children and adolescents with IR, while none of the participants was diagnosed with T2DM (Table 2). Children with IR were primarily girls (*P*=0.035); in addition, significantly more preterm birth and higher Tanner stages were observed in the IR group (Tables 1 and 2). No differences were observed in blood pressure, waist circumference, and family history. In adolescents with IR, an obese waist circumference was significantly more often observed (Table 2). They also had significantly more family members with dyslipidemia ((first degree 32 (15.2%) versus 22 (10.3%), second degree (60 (28.4%) versus 42 (19.7%)), *P*=0.015)). Furthermore, systolic blood pressure was significantly higher (Table 2). No differences were found in family history of T2DM, obesity, hypertension, and CVDs.

The multivariate logistic regression model showed that term of birth, Tanner stage, and BMI-SDS were associated with IR development in children. In adolescents, BMI-SDS and waist circumference were associated with IR (Table 4).

## 4. Discussion

Obesity is increasingly prevalent at younger age and so are related complications such as MetS and T2DM [1–3]. Since IR is associated with obesity and a known risk factor for MetS and T2DM, recognizing the predictors of IR is crucial for optimal screening. Different predictors of IR in children and adolescents with obesity were observed. BMI-SDS was associated with IR in children and adolescents. Furthermore, preterm birth and Tanner stage were associated with IR in children, and waist circumference was associated with IR in adolescents.

Influence of puberty on IR development is a much debated topic. Cross-sectional studies have shown that the prevalence of IR is increasing at start of puberty, peaks at Tanner stage 3, and returns to prepubertal levels by the end of puberty, regardless of the presence of obesity [10, 15, 16]. It is known that transient physiological IR induces extra stress on pancreatic beta cells, which makes puberty a risky period for MetS and/or T2DM development, especially in those with obesity [10].

In the current study, an association between IR and Tanner stage was only observed in children. This suggests that IR in children is influenced by puberty in contrast to adolescents. In addition, early onset of puberty is associated with increased cardiometabolic risk, at least in girls [27]. This emphasizes that screening on IR in children with early onset of puberty is essential. An explanation for the observed difference in the association with puberty in children and adolescents could be the degree of obesity in the adolescent population, as obesity contributes to IR development [6, 7, 16, 28]. In the current study, the degree of obesity, especially the waist circumference as measure of fat distribution in the adolescent population, may blur the association with puberty. This may explain why Tanner stage is not a predictor of IR in adolescents, and therefore, screening during puberty in adolescents is warranted.

The importance of waist circumference in defining obesity instead of BMI-SDS is also emphasized in recent literature [8, 23, 29, 30]. In the studied population, only in adolescents, waist circumference was associated with IR. This stresses the importance of measuring waist circumference during routine clinical care. Why waist circumference is not associated with IR in children could be the effect of the different fat distribution [16, 28]. Goran et al. assumed that lack of major effects of abdominal fat mass on insulin sensitivity in young children may be explained by relatively lower levels of subcutaneous abdominal fat [15]. Based on our data, waist circumference might not be the preferable method to define obesity in children as discussed in recent literature. Therefore, in children, BMI-SDS should be used to define obesity.

The observation that IR was associated with preterm birth, supported by studies describing a relationship between preterm birth and altered insulin homeostasis, is demonstrated by an increased risk of IR in childhood and young adulthood [18, 31–33]. Special attention for IR during long-term follow-up of preterm-born children is therefore recommended. In adolescents no significant association between IR and preterm brith was observed, although a trend was shown. Possibly other factors may have a greater influence, or missing data influenced the results.

In children, girls were significantly more diagnosed with IR. This is in concordance with recent literature; in addition, a higher prevalence of IR in girls without obesity is described [13]. This result can be explained by the fact that pubertal development starts earlier in girls. In adolescents, no difference in gender was observed, possibly because the degree of obesity blurred the effect of puberty as described earlier.

Obesity is a well-known risk factor for MetS and T2DM [12]. MetS was frequently diagnosed in children and adolescents with IR. T2DM was not yet diagnosed in the studied population; however, many participants already had an IGT. These observations are worrisome, since it is known that children and adolescents with obesity have a risk up to 70% to remain obese in adulthood [34]. Abdominal fat is associated with a higher systolic blood pressure [35]. The high systolic blood pressure observed in adolescents with IR is probably influenced by the level of subcutaneous fat mass, as an obese waist circumference was more frequently measured in the adolescents with IR. This might explain as well, why in children this difference was not observed. Higher triglyceride and lower HDL levels were observed in children and adolescents with IR. The association with IR and alterations in lipid profile is in line with the literature, although no discrepancy was made between children and adolescents [30, 34]. Based on the association with IR, the suggestion to incorporate triglycerides in the definition of IR is done [36, 37]; however, added value has not yet been proven [13].

This study examined systematically the predictors of IR in a large cohort of children and adolescents with obesity in the Netherlands. We focused on BMI-SDS and other known risk factors for development of IR. However, certain limitations due to the retrospective cross-sectional observational study design must be considered. Firstly, the intraindividual variation in the measurement of plasma insulin has to be mentioned. The intraindividual variation is caused by the pulsatile excretion of insulin which is influenced by the time of the blood sample and the condition of the patient, especially stressed and/or active. Variation up to 12% in the plasma insulin concentration and consequently in the HOMA-IR has been described [26, 38]. Therefore, to minimize this, intraindividual variation in insulin measurements should be performed twice under standardised circumstances. All fasted blood samples were taken at the pediatric day clinic, early morning by vena puncture after an overnight fasting period. Although insulin measurements have been performed, according to the hospital protocol, intraindividual variation might have influenced the prevalence of IR.

Secondly, the uniform cutoff value of the HOMA-IR to diagnose IR in the entire study population might have influenced the prevalence of IR. Since the used cutoff value was based on the mean value for 95th percentile in two studies in normal weight children with a similar age range, we think that the uniform cutoff value for HOMA-IR was justified [26, 39, 40]. Finally, consequently to retrospective studies, incomplete data exist. In the current study, incomplete data were observed on medical history (e.g., pregnancy, neonatal, and weight gain in the first year of life) and sedentary life style. The incomplete data might have had some impact on the study results. Therefore in prospective studies, data on medical history and sedentary life style are obligatory to study the impact on the risk factors of IR in children and adolescents with obesity.

## 5. Conclusions

In the current study, different predictors of IR were observed in children and adolescents. BMI-SDS was associated with IR in children and adolescents, preterm birth and Tanner stage were associated with IR in children, and waist circumference was associated with IR in adolescents. These predictors can be used to optimize screening for IR in pediatric populations. In future prospective studies, the impact of medical history and sedentary life style on the risk factors of IR in children and adolescents with obesity should be studied.

## Figures and Tables

**Table 1:** Demographics of obese children and adolescents, stratified by IR.

	Children *n* = 306			Adolescents *n* = 471		
	IR *n* = 51	No IR *n* = 255	*P* value	IR *n* = 233	No IR *n* = 238	*P* value
Age (yr)	8.1 (3.9–9.9)	7.3 (2.4–9.9)	**0.003**	13.2 (10.0–17.9)	12.3 (10.0–17.8)	**0.001**
Male *n* (%)	16 (31.4)	121 (47.5)	**0.035**	115 (49.4)	126 (52.9)	0.436
Caucasian *n* (%)	35 (68.6)	167 (65.5)	0.829	157 (67.4)	137 (57.6)	0.128
Preterm birth *n* (%)	8 (15.6)	16 (6.3)	**0.027**	21 (9.0)	13 (5.5)	0.224
SGA/LGA *n* (%)	8 (15.6)	34 (13.3)	0.708	38 (16.3)	46 (19.3)	0.141
Breastfeeding *n* (%)	17 (33.3)	77 (30.2)	0.709	83 (35.6)	75 (31.5)	0.170

Data presented as number (%), mean (standard deviation), or median with range. SGA/LGA = small for gestational age/large for gestational age. *P* values for unpaired *t*-test (normally distributed data), Mann–Whitney *U* test (nonparametric data), and χ^2^ tests (categorical data). *P* values in bold are significant at <0.05.

**Table 2:** Anthropometric measurements of obese children and adolescents, stratified by IR.

	Children *n* = 306			Adolescents *n* = 471		
	IR *n* = 51	No IR *n* = 255	*P* value	IR *n* =233	No IR *n* = 238	*P* value
Length (cm)	134.1 (10.9)	128.3 (13.6)	**0.004**	162.2 (10.5)	158.5 (11.9)	**<0.001**
Length-SDS	0.64 (1.04)	0.47 (0.99)	0.280	0.16 (1.05)	0.10 (1.09)	0.569
Weight (kg)	47.9 (11.6)	40.2 (10.7)	**<0.001**	85.2 (20.5)	75.2 (17.9)	**<0.001**
BMI (kg/m^2^)	26.3 (3.2)	24.1 (2.7)	**<0.001**	31.9 (5.0)	29.4 (3.8)	**<0.001**
BMI-SDS	3.70 (0.69)	3.41 (0.77)	**0.015**	3.24 (0.47)	3.01 (0.46)	**<0.001**
Obese waist *n* (%)	29 (56.9)	141 (55.2)	0.557	144 (61.8)	124 (52.1)	**0.002**
High SBP *n* (%)	23 (45.1)	92 (36.0)	0.193	102 (43.8)	76 (31.9)	**0.022**
High DBP *n* (%)	6 (11.8)	34 (13.3)	0.759	21 (9.0)	17 (7.1)	0.555
Tanner stage *n* (%)	—	—	**<0.001**	—	—	0.550
(i) Prepubertal (T1)	38 (74.5)	232 (90.9)	—	37 (15.9)	46 (19.3)	—
(ii) Pubertal (T2–4)	11(21.6)	12 (4.7)	—	108 (45.4)	102 (42.9)	—
(iii) Postpubertal (T5)	—	—	—	39 (16.7)	37 (15.5)	—
MetS *n* (%)	13 (25.5)	25 (9.8)	**0.002**	71 (30.5)	24 (10.1)	**<0.001**
T2DM *n* (%)	—	—	—	—	—	—

Data presented as number (%), mean (standard deviation), or median with range. BMI = body mass index, SDS = standard deviation score, SBP = systolic blood pressure, DBP = diastolic blood pressure, MetS = metabolic syndrome, T2DM = type II diabetes mellitus. *P* values for unpaired *t*-test (normally distributed data), Mann–Whitney *U* test (nonparametric data), and χ^2^ tests (categorical data). *P* values in bold are significant at <0.05.

**Table 3:** Laboratory results of obese children and adolescents, stratified by IR.

	Children *n* = 306			Adolescents *n* = 471		
	IR *n* = 51	No IR *n* = 255	*P* value	IR *n* = 233	No IR *n* = 238	*P* value
FPG (mmol/L)	5.1 (0.5)	4.9 (0.4)	**<0.001**	5.2 (0.5)	5.0 (0.4)	**<0.001**
FPI (mU/L)	19.5 (11.0–50.2)	5.4 (1.0–16.0)	**<0.001**	24.0 (13.9–69.0)	9.0 (1.1–15.7)	**<0.001**
HOMA-IR	4.38 (3.4–13.4)	1.16 (0.2–3.3)	**<0.001**	4.85 (3.4–19.9)	1.99 (0.3–3.3)	**<0.001**
TC (mmol/l)	4.30 (0.73)	4.32 (0.74)	0.716	4.3 (0.85)	4.3 (0.77)	0.949
HDL (mmol/l)	1.2 (0.34)	1.3 (0.30)	**0.039**	1.15 (0.26)	1.27 (0.29)	**<0.001**
LDL (mmol/l)	2.6 (0.71)	2.49 (0.62)	0.291	2.6 (0.75)	2.5 (0.69)	0.525
TG (mmol/l)	1.4 (0.46)	0.9 (0.63)	**<0.001**	1.29 (0.66)	1.04 (0.54)	**<0.001**

Data presented mean (standard deviation) or median with range. FPG = fasting plasma glucose, FPI = fasting plasma insulin, HOMA-IR = homeostatic assessment for IR, IR = insulin resistance, TC = total cholesterol, TG = triglycerides. *P* values for unpaired *t*-test (normally distributed data) and Mann–Whitney *U* test (nonparametric data). *P* values in bold are significant at <0.05.

**Table 4:** Adjusted^∗^ RRs and 95% CI between BMI-SDS, Tanner stage, term of birth, birth weight, breastfeeding, and waist circumference (independent variables) and IR (dependent variable).

	Children		Adolescents	
	RR (95% CI)	*P* value	RR (95% CI)	*P* value
BMI-SDS	2.19 (1.01–4.76)	**0.048**	3.96 (1.70–9.21)	**0.001**
Tanner stage	5.43 (1.44–20.45)	**0.012**	1.70 (0.85–3.41)	0.137
Term of birth	6.29 (1.27–31.05)	**0.024**	2.64 (0.54–12.83)	0.230
Birth weight	1.66 (0.36–7.61)	0.513	0.49 (0.21–1.13)	0.093
Breastfeeding	1.72 (0.61–4.86)	0.309	1.58 (0.76–3.29)	0.222
Waist circumference	1.15 (0.30–4.37)	0.840	3.743 (1.49–9.36)	**0.005**

^∗^Adjustment for age, gender, and ethnicity. *P* values in bold are significant at <0.05.

## References

[1] World Health Organisation Global and regional trends by UN Regions, 1990–2025; Overweight: 1990–2015. http://apps.who.int/gho/data/node.main.NUTUNREGIONS?lang=en.

[2] Lobstein T., Jackson-Leach R., Moodie M. L. (2015). Child and adolescent obesity: part of a bigger picture. *The Lancet*.

[3] de Onis M., Blossner M., Borghi E. (2010). Global prevalence and trends of overweight and obesity among preschool children. *American Journal of Clinical Nutrition*.

[4] Han J. C., Lawlor D. A., Kimm S. Y. (2010). Childhood obesity. *The Lancet*.

[5] Kelly A. S., Barlow S. E., Rao G. (2013). Severe obesity in children and adolescents: identification, associated health risks, and treatment approaches: a scientific statement from the American Heart Association. *Circulation*.

[6] Castro A. V., Kolka C. M., Kim S. P., Bergman R. N. (2014). Obesity, insulin resistance and comorbidities? Mechanisms of association. *Arquivos Brasileiros de Endocrinologia and Metabologia*.

[7] Kahn S. E., Hull R. L., Utzschneider K. M. (2006). Mechanisms linking obesity to insulin resistance and type 2 diabetes. *Nature*.

[8] Lee J. M. (2006). Insulin resistance in children and adolescents. *Reviews in Endocrine and Metabolic Disorders*.

[9] Levy-Marchal C., Arslanian S., Cutfield W. (2010). Insulin resistance in children: consensus, perspective, and future directions. *Journal of Clinical Endocrinology and Metabolism*.

[10] Kurtoglu S., Hatipoglu N., Mazicioglu M., Kendirici M., Keskin M., Kondolot M. (2010). Insulin resistance in obese children and adolescents: HOMA-IR cut-off levels in the prepubertal and pubertal periods. *Journal of Clinical Research in Pediatric Endocrinology*.

[11] Steinberger J., Daniels S. R., American Heart Association Atherosclerosis, Hypertension, and Obesity in the Young Committee (Council on Cardiovascular Disease in the Young), American Heart Association Diabetes Committee (Council on Nutrition, Physical Activity, and Metabolism) (2003). Obesity, insulin resistance, diabetes, and cardiovascular risk in children: an American Heart Association scientific statement from the Atherosclerosis, Hypertension, and Obesity in the Young Committee (Council on Cardiovascular Disease in the Young) and the Diabetes Committee (Council on Nutrition, Physical Activity, and Metabolism). *Circulation*.

[12] Alberti K. G., Eckel R. H., Grundy S. M. (2009). Harmonizing the metabolic syndrome: a joint interim statement of the International Diabetes Federation Task Force on Epidemiology and Prevention; National Heart, Lung, and Blood Institute; American Heart Association; World Heart Federation; International Atherosclerosis Society; and International Association for the Study of Obesity. *Circulation*.

[13] van der Aa M. P., Fazeli Farsani S., Knibbe C. A., de Boer A., van der Vorst M. M. (2015). Population-based studies on the epidemiology of insulin resistance in children. *Journal of Diabetes Research*.

[14] van der Aa M. P., Knibbe C. A., Boer A., van der Vorst M. M. (2017). Definition of insulin resistance affects prevalence rate in pediatric patients: a systematic review and call for consensus. *Journal of Pediatric Endocrinology and Metabolism*.

[15] Goran M. I., Gower B. A. (2001). Longitudinal study on pubertal insulin resistance. *Diabetes*.

[16] Moran A., Jacobs D. R., Steinberger J. (1999). Insulin resistance during puberty: results from clamp studies in 357 children. *Diabetes*.

[17] Ling J. C., Mohamed M. N., Jalaludin M. Y., Rampal S., Zaharan N. L., Mohamed Z. (2016). Determinants of high fasting insulin and insulin resistance among overweight/obese adolescents. *Scientific Reports*.

[18] Hofman P. L., Regan F., Jackson W. E. (2004). Premature birth and later insulin resistance. *New England Journal of Medicine*.

[19] Sabin M. A., Magnussen C. G., Juonala M. (2015). Insulin and BMI as predictors of adult type 2 diabetes mellitus. *Pediatrics*.

[20] Figueroa Sobrero A., Evangelista P., Kovalskys I. (2016). Cardio-metabolic risk factors in Argentine children. A comparative study. *Diabetes and Metabolic Syndrome: Clinical Research and Reviews*.

[21] Visser G. H., Eilers P. H., Elferink-Stinkens P. M., Merkus H. M., Wit J. M. (2009). New Dutch reference curves for birthweight by gestational age. *Early Human Development*.

[22] TNO De TNO groeicalculator voor professionals-op basis van de vijfde landelijke groeistudie. https://www.tno.nl/nl/aandachtsgebieden/gezond-leven/prevention-work-health/gezond-en-veilig-opgroeien/groeicalculator-voor-professionals/.

[23] Fredriks A. M., van Buuren S., Fekkes M., Verloove-Vanhorick S. P., Wit J. M. (2005). Are age references for waist circumference, hip circumference and waist-hip ratio in Dutch children useful in clinical practice?. *European Journal of Pediatrics*.

[24] National High Blood Pressure Education Program Working Group on High Blood Pressure in Children and Adolescents (2004). The fourth report on the diagnosis, evaluation, and treatment of high blood pressure in children and adolescents. *Pediatrics*.

[25] Matthews D. R., Hosker J. P., Rudenski A. S., Naylor B. A., Treacher D. F., Turner R. C. (1985). Homeostasis model assessment: insulin resistance and beta-cell function from fasting plasma glucose and insulin concentrations in man. *Diabetologia*.

[26] van der Aa M. P., Fazeli Farsani S., Kromwijk L. A., de Boer A., Knibbe C. A., van der Vorst M. M. (2014). How to screen obese children at risk for type 2 diabetes mellitus?. *Clinical Pediatrics*.

[27] Boyne M. S., Thame M., Osmond C. (2014). The effect of earlier puberty on cardiometabolic risk factors in Afro-Caribbean children. *Journal of Pediatric Endocrinology and Metabolism*.

[28] Gobato A. O., Vasques A. C., Zambon M. P., de Azevedo Barros Filho A., Hessel G. (2014). Metabolic syndrome and insulin resistance in obese adolescents. *Revista Paulista de Pediatria*.

[29] Gonzalez-Jimenez E., Schmidt-RioValle J., Montero-Alonso M. A., Padez C., Garcia-Garcia C. J., Perona J. S. (2016). Influence of biochemical and anthropometric factors on the presence of insulin resistance in adolescents. *Biological Research For Nursing*.

[30] Ali O., Cerjak D., Kent J. W., James R., Blangero J., Zhang Y. (2014). Obesity, central adiposity and cardiometabolic risk factors in children and adolescents: a family-based study. *Pediatric Obesity*.

[31] Tinnion R., Gillone J., Cheetham T., Embleton N. (2014). Preterm birth and subsequent insulin sensitivity: a systematic review. *Archives of Disease in Childhood*.

[32] Sipola-Leppanen M., Vaarasmaki M., Tikanmaki M. (2015). Cardiometabolic risk factors in young adults who were born preterm. *American Journal of Epidemiology*.

[33] Wang G., Divall S., Radovick S. (2014). Preterm birth and random plasma insulin levels at birth and in early childhood. *Journal of the American Medical Association*.

[34] dos Santos Romualdo M. C., de Nóbrega F. J., Escrivão M. A. M. S. (2014). Insulin resistance in obese children and adolescents. *Jornal de Pediatria*.

[35] Jansen M. A., Uiterwaal C. S., Visseren F. L., van der Ent C. K., Grobbee D. E., Dalmeijer G. W. (2016). Abdominal fat and blood pressure in healthy young children. *Journal of Hypertension*.

[36] Love-Osborne K., Butler N., Gao D., Zeitler P. (2006). Elevated fasting triglycerides predict impaired glucose tolerance in adolescents at risk for type 2 diabetes. *Pediatric Diabetes*.

[37] McAuley K. A., Williams S. M., Mann J. I. (2001). Diagnosing insulin resistance in the general population. *Diabetes Care*.

[38] Henriquez S., Jara N., Bunout D. (2013). Variability of formulas to assess insulin sensitivity and their association with the Matsuda index. *Nutrición hospitalaria*.

[39] Allard P., Delvin E. E., Paradis G. (2003). Distribution of fasting plasma insulin, free fatty acids, and glucose concentrations and of homeostasis model assessment of insulin resistance in a representative sample of Quebec children and adolescents. *Clinical Chemistry*.

[40] d’Annunzio G., Vanelli M., Pistorio A. (2009). Insulin resistance and secretion indexes in healthy Italian children and adolescents: a multicentre study. *Acta Biomedica*.

